# Building Towards Initiation, Moderation, De-Escalation and Cessation of Disease-Modifying Treatments for Multiple Sclerosis in Greece: An Expert Panel Consensus Meeting

**DOI:** 10.3390/brainsci16060580

**Published:** 2026-05-29

**Authors:** Marina Kleopatra Boziki, Christos Bakirtzis, Harry Alexopoulos, Efthimios Dardiotis, Maria-Eleftheria Evangelopoulos, Sotirios Giannopoulos, Vasiliki Kostadima, Evangelos Kouremenos, Panos Stathopoulos, Vaia Tsimourtou, Dimitrios Tzanetakos, Ioannis Iliopoulos, Nikolaos Grigoriadis

**Affiliations:** 1 Multiple Sclerosis Center, 2nd Neurological University Department, AHEPA University Hospital, Aristotle University of Thessaloniki, 54636 Thessaloniki, Greece; bozikim@auth.gr (M.K.B.); cbakirtzis@auth.gr (C.B.); 2 Department of Cell Biology & Biophysics, Faculty of Biology, National and Kapodistrian University of Athens, 15701 Athens, Greece; halexo@gmail.com; 3 Neurology Clinic, University General Hospital of Larissa, University of Thessaly, 41334 Larissa, Greece; edar@uth.gr (E.D.); vana.tsimourtou@gmail.com (V.T.); 4 Demyelinating Disorders Clinic, 1st Department of Neurology, Aiginition Hospital, School of Medicine, National and Kapodistrian University of Athens, 11528 Athens, Greece; evangelopoulos@yahoo.com (M.-E.E.); pmstathopoulos@gmail.com (P.S.); 5 Second Department of Neurology, Attikon University Hospital, National and Kapodistrian University of Athens, 12462 Athens, Greece; sgianno@med.uoa.gr (S.G.); tzanetakosdim@yahoo.com (D.T.); 6 Department of Neurology, University Hospital of Ioannina, Faculty of Medicine, University of Ioannina, 45500 Ioannina, Greece; veniakostadima@gmail.com; 7 Neurology Department, 251 Hellenic Air Force General Hospital, 11525 Athens, Greece; vkoure@otenet.gr; 8 Neurology Department, University General Hospital of Alexandroupolis, Democritus University of Thrace, 68100 Alexandroupolis, Greece; iiliop@med.duth.gr

**Keywords:** relapsing multiple sclerosis, disease-modifying treatment, treatment switch, disease management, personalized treatment

## Abstract

**Background/Objectives:** Multiple Sclerosis (MS) is a chronic disease with significant clinical and radiological heterogeneity. This fact, together with the increased number of disease-modifying treatments available, poses challenges in the therapeutic decisions and for the overall management of the disease. In this study, an expert panel on MS from Greece aimed to formulate a consensus, in order to provide recommendation on disease-modifying treatment (DMT) initiation and switching, as well as de-escalation strategies in Relapsing MS (RMS). **Methods**: The study followed two-round voting based on a modified Delphi setting. A questionnaire was constructed by a subgroup of five experts (core group) and was subsequently administered in a printed form to a group of 12 MS experts in total (panel) in a face-to-face meeting. Consensus required at least 80% agreement within the panel in order to signify strong consensus. **Results:** The panel agreed that the overall therapeutic plan (DMT choice) must take into consideration the degree of disease activity (low/moderate/high). In certain cases with suboptimal response to a moderate-efficacy DMT, a horizontal switch to another moderate-efficacy DMT may be a valid strategy. However, in cases exhibiting disability accumulation, therapy escalation should be preferred. The concept of de-escalation was suggested as an alternative strategy for cases with stable disease receiving a high-efficacy long-term DMT in the long term. Due to the possibility of rebound phenomena with certain medications (such as fingolimod and natalizumab), a bridging strategy could be applied in cases of family planning and drug-related adverse events (such as lymphopenia and hepatotoxicity), especially in PwMS with recent inflammatory activity. **Conclusions:** Although novel biomarkers may soon help clinicians predict future disability accumulation, currently, regular and detailed patient monitoring seems to be the optimal way to guide clinicians’ decisions on treatment changes.

## 1. Introduction

Multiple Sclerosis (MS) is a chronic disease with significant clinical and radiological heterogeneity. This fact, together with the increased number of disease-modifying treatments available, it poses challenges in therapeutic decisions and for the overall management of the disease. In Multiple Sclerosis (MS), the need for personalized treatment approaches has been elucidated. However, biomarkers for disease prognosis and/or prediction of treatment response are currently lacking. Although extensive research on advanced imaging and/or laboratory techniques have identified potential markers that depict several aspects of disease pathology [i.e., gray matter pathology, neurofilament light chain (NfL), etc.] [1,2], these measurements have not been adequately validated in a setting that would, at least currently, allow for their integration in clinical decision-making. Disease-modifying treatments (DMTs) are characterized by variability in their mode of action and patient-related factors, such as age, comorbidities and preferences, that warrant careful benefit–risk assessment prior to treatment selection [3,4]. Guidelines for treatment management in MS [5,6,7] exist, as well as expert group statements [8,9]. However, several issues related to DMT switching remain largely dependent on the physicians’ judgment and the Centers’ practice.

The administrative prevalence of MS in Greece has recently been estimated on the basis of a Nationwide Prescription Database. The 2-year cumulative prevalence of MS was estimated as 197.8 per 100,000, with >21,000 patients identified [10]. In this study, the prevalence of MS reported in Greece was comparable to the prevalence reported for other European countries. Moreover, the reported change in age distribution over time toward gradually increasing age at the prevalence peak has also been described for other European populations and underlines the increasing mean age of the patients’ cohorts in the frame of improved overall mortality rates for people with MS (PwMS) [11]. As MS is a chronic disease and thus MS patients are expected to receive DMTs for several years following diagnosis, optimization of treatment management and physicians’ support with guidelines and treatment recommendations that answer common issues anticipated in everyday clinical practice remains imperative.

In this study, an expert panel on MS from Greece aimed to formulate a consensus, in order to provide recommendation on DMT initiation and switching, as well as de-escalation strategies in Relapsing MS (RMS).

## 2. Materials and Methods

### 2.1. Study Design

This is a Consensus study, following two-round voting [12] and based on a modified Delphi setting. Both round-meetings were conducted in a face-to-face setting in order to facilitate direct communication and discussion. The questionnaire-based approach was selected in order to facilitate participation in a transparent and reproducible manner [13], as measures to minimize bias. By taking into account the broad spectrum of the developed recommendations that cover several aspects of MS management, a two-round, structured questionnaire-based approach was followed as a cost-effective procedure to reach consensus and to elucidate topics in need of further clarification. A questionnaire was constructed by a subgroup of five experts (core group) and was subsequently administered in a printed form to a group of 12 MS experts in total (panel), in a face-to-face meeting. Following open discussion, panel members anonymously voted “agree” or “disagree” in written form on the questionnaire for each question. In this respect, although opinions during the open discussion were not formulated anonymously, thus posing, at least in part, potential bias, the final in-paper recording of the answers was anonymous. Consensus required at least 80% agreement within the panel in order to signify strong consensus, as previously described [14,15,16]. Statements with less than 80% agreement were modified by the core panel, based on an open discussion and by taking into account feedback provided by the panel. These statements were then re-circulated for voting. Following this, statements not reaching 80% agreement were further discussed and are hereby reported but are not included in the final consensus statement. Due to the fact that the present manuscript reports experts’ opinions, review and approval of a Bioethics’ Committee is non-applicable and, therefore, was not conducted. The results from the consensus meeting are hereby reported in detail.

### 2.2. Panel Selection

Twelve neurologists, MS experts from across Greece, were invited based on their clinical and academic profile as a professional group with long-term expertise in the care and management of people with MS. Expert selection criteria included >10 years of experience in treating PwMS, as evidenced by working in a specialized health setting that allows experience with the whole spectrum of DMTs for MS. Examples include specialized clinic departments and/or outpatient practices, as well as day-care facilities in tertiary hospitals. In addition to expertise in the field, interest was taken in order to fulfill the criterion of geographical spread upon panel member selection [17]. In addition to the fact that all experts had significant experience with the whole spectrum of DMTs for MS, the study was organized on a strictly scientific setting with all statements formulated in relevance to treatment categories instead of individual treatments, aiming to reduce potential conflicts of interest. All experts were asked to provide a conflict of interest form, as means to ensure transparency.

### 2.3. Statements

A PubMed search between July 2020 and July 2023 by the terms: “multiple sclerosis”; “disease-modifying treatment”; “relapsing multiple sclerosis”, “disease management”, “treatment switch” and “consensus” was the initial step. Earlier pivotal DMT trials and guidelines from international organizations such as ECTRIMS were additionally studied. Based on the literature review the core group focused on five topics (chapters): (I) treatment aims; (II) suboptimal treatment response; (III) practice of treatment change; (IV) reasons for treatment change; (V) statements for injectable immunomodulatory drugs and bridging therapy. The derived statements were evaluated by the expert panel over two rounds of voting. In the event that a statement consensus was not reached, the statement was (a) discarded, (b) rephrased and submitted for voting over the second round, or (c) divided into two more detailed statements and submitted for voting over the second round. The specific strategy was suggested by the core panel on the basis of the arguments made during the first round. In case of disagreements, respective evidence was documented and the statement was rephrased accordingly in order to be submitted in the second-round voting, when possible. The statements that reached consensus either in both rounds or only the second round are presented below the description of each chapter in respective tables. Rejected or negatively agreed statements are presented in Supplementary Table S1.

## 3. Results

For the first round, consensus, either positive or negative (agreement of at least 80% of participants) was reached in 53/59 statements (90%), while 3/59 (5%) were discarded due to lack of agreement. Moreover, 3/59 statements (5%) were separated into two different statements and further submitted to the second round. Therefore, after the second round, consensus, either positive or negative, was reached in 59/59 responses (100%).

### 3.1. Topic I: Treatment Aims

Disease activity is defined as the presence of relapses and/or the presence of new/enlarged T2 MRI lesions. Disease activity in MS is considered to depict active neuroinflammation in the Central Nervous System (CNS) [18,19]. A relapse (or a demyelinating episode) is defined as a monophasic clinical episode with new patient-reported symptoms and objective findings in the neurological examination, reflecting focal or multifocal pathology in the CNS. The typical temporal evolution of a relapse is to develop acutely or, more frequently, subacutely and lasting to a minimum of 24 h. A relapse may be followed by full, partial or no recovery, and it must occur in the absence of fever or infection [20,21]. The presence of relapses in a patient receiving DMT for MS indicates ongoing disease activity and poses concerns on the DMT efficacy. Disease activity (either clinical or radiological) constitutes the main outcome for the selection of the first-choice DMT and the subsequent ones. Regarding pseudo-relapses, the panel agreed that a pseudo-relapse may result upon a condition of stress, a fever, an infection, etc., for a person with MS. A pseudo-relapse is the increased severity of a pre-existing neurological symptom that occurs in the presence of, among other causes, infection, exposure to increased external temperature, fatigue, stress, or dehydration. Pseudo-relapses are often attributed to the Uhthoff phenomenon, which refers to a transient worsening of neurological function lasting less than 24 h that can occur in PwMS and is attributed to the transient conduction dysfunction of previously damaged myelinated fibers due to increases in core body temperature. Similarly, a confirmed Uhthoff phenomenon usually excludes relapse [22]. The distinction between relapses and pseudo-relapses is crucial, as pseudo-relapses are treated by eliminating the triggering factor. Rest and hydration, as well as treatment of an underlying infection, are such measures. In this respect, steroids are not an appropriate treatment for the management of pseudo-relapse symptoms.

The panel further discussed on the definition of a relapse. In order to confirm a relapse, the physician should be able to witness a change in the Expanded Disability Status Scale (EDSS) [23] and in the other scales routinely used for disability assessment. Although the EDSS provides a semi-quantitative assessment of the MS-related disability, usually certain changes in the sub-scales (assessing separately functional system scores) are expected to affect the total EDSS. This means that a ΔEDSS of 0.5 or 1 may signify a relapse, provided that there is an absence of fever, infection or other pseudo-relapse-associated factors preceding [22]. The panel agreed that a relapse is signified by “new” signs/symptoms and not the transient exacerbation of previously existing signs/symptoms for a brief period of <24 h.

Regarding treatment objectives and strategies, the panel agreed that the overall therapeutic plan (DMT choice) must take into consideration the degree of disease activity (low/moderate/high). Although no universal consensus exists on the definition of high disease activity, the more widely accepted definition is that of patients with at least two relapses and/or patients with at least nine T2 lesions and/or one enhancing T1 lesion within a 12-month period [5,7]. Overall, patients with high disease activity are exhibiting frequent relapses (short inter-attack interval) and/or increased burden of radiological activity on MRI either as new/enlarging T2 lesions or as the presence of gadolinium-enhancing lesions (Gd+). Additional characteristics include increased severity of relapses, possible lesion localization in sites linked with poor prognosis, such as infratentorial and/or the spinal cord, and ongoing clinical or MRI disease activity while already on a DMT prophylactic treatment. Incomplete relapse recovery and faster accumulation of fatigue or cognitive impairment may also be observed in PwMS with high disease activity [24].

In this respect, the panel agreed that the definition of ‘high activity’, also included in the Summary of Product Characteristics (SPC) of several high-efficacy DMTs, needs to be redefined in order to follow the current clinical practice. Moreover, the panel agreed that the treatment objective should be the control of disease activity in order to prevent relapses, including the prevention of disability worsening and thereby prevention or delay of disability progression, across the disease course. The panel discussed that the treatment aims to control CNS inflammation to avoid putative relapses and/or to prevent or delay disability worsening/deterioration. The panel agreed that a No Evidence of Disease Activity-3 (NEDA-3) status, defined as no new/enlarging T2 weighted lesions/Gd+ lesions on MRI of the brain, no new clinical relapses, and no confirmed worsening of EDSS [25], is a desirable outcome; however, the panel considers that in some cases, this objective may be difficult to reach in the long term [26]. Therefore, the panel agreed that Minimal Evidence of Disease Activity (MEDA) may constitute a more realistic clinical outcome than NEDA for the assessment of the optimal clinical response to DMTs. Several definitions of MEDA have been proposed, but without a universally accepted consensus. Rio et al. defined MEDA as either the presence of <3 new T2 lesions or <2 Gd+ lesions in the absence of clinical activity, or as the presence of one relapse with 0 or 1–2 new T2 lesions [27]. Isolated minimal clinical activity with one relapse that is not associated with a clinical sequel was also described under the definition of MEDA, and MEDA as an outcome was not related with EDSS increase in the long term. In a study by Sormani et al., isolated minimal MRI activity defined by the absence of relapse and the presence of ≤2 new T2 MRI lesions was also not associated with EDSS worsening [28]. In this respect, MEDA includes all PwMS who exhibit NEDA as well as those presenting minimal MRI activity, but otherwise clinically stable disease [29,30,31]. In line with the existing evidence, the panel agreed that MEDA could be defined as the absence of relapses or disability progression and minimal (≤T2 lesions) MRI activity (Rio score 0). Although MEDA is a promising outcome, exhibiting reproducible association with favorable MS prognosis in terms of EDSS worsening in the long term, few studies have evaluated MEDA in the real-world setting. Therefore, MEDA as an outcome poses a considerable limitation in its clinical application, depicted in the relatively small number of studies in the literature that elaborate on this tool, as means to address disease prognosis. Regarding the time following treatment initiation during which a relapse may be experienced by the patient, usually it is during the initial 6 months following treatment initiation. This period accounts for a “grace period”, further from which relative treatment failure should be considered.

The panel discussed whether Progression Independent of Relapse Activity (PIRA) is a reasonable and important clinical outcome to consider for treatment choice. PIRA and Relapse-Associated Worsening (RAW) are patterns of irreversible disability accumulation in MS and may occur at any stage across the disease course [32,33,34,35].

The panel did not agree that PIRA is a reasonable and important clinical outcome to consider for treatment choice. Considerations were expressed regarding the fact that PIRA has not yet been studied for all drugs available. Although PIRA as an outcome is included in NEDA, the majority of available treatment options target disease activity.

The panel agreed that early initiation of prophylactic treatment as soon as MS diagnosis is made is a necessity. However, the panel agreed that a DMT should not be offered in an elderly, treatment-naïve patient without sings of disease activity. The age that the panel suggested as a cut off for the initiation of a DMT is >65 years of age, but this should be re-evaluated on a personalized basis. The panel agreed on the early initiation of treatment. However, in accordance with the fact that patient engagement is a crucial component in the modern clinical setting, additional time may be given to the patient until actual treatment initiation for few months, on the basis of a patient’s informed decision (even 5–6 months after diagnosis), based on current disease activity, disability, MRI lesion localization, patient’s current life status and preferences. Topic I statements are presented in Table 1.

### 3.2. Topic II: Suboptimal Treatment Response

The panel agreed that suboptimal response to treatment must be defined on the basis of the presence of clinical activity (relapses and/or increase in disability, relapse dependent or independent, and/or radiological activity (as defined with two or more active lesions). Moreover, the panel agreed that the term of suboptimal response is applicable for all DMTs across the disease course; however, the initial evaluation of response to treatment should be 6–12 months following to treatment initiation, depending on the DMT mechanism of action (in some DMTs re-baseline could be performed 3 months after initiation, while in most of the DMTs, re-baseline should be performed after 6 months of treatment). This time of evaluation may be different for DMTs with a longer biological effect, for instance, cladribine. For cladribine, a more extended time period for evaluation should be applied, that is, 24 months, in order for the treatment to be considered as complete [36,37,38]. The panel agreed that response to a DMT administered as short treatment cycle (corresponding to immune reconstitution treatments/IRTs cladribine and alemtuzumab) should be better assessed once all cycles have been administered. The panel agreed that the currently existing evidence does not suffice to justify the inclusion of other outcomes such as cognitive performance, metrics such as retinal nerve fiber layer (RNFL) in optical coherence tomography (OCT), serum biomarkers such as levels of serum NfL, and MRI metrics such as rates of brain volume loss as parameters to assess therapeutic response. However, upon open discussion, the panel acknowledged the existing evidence based on extensive research that underlines the potential of the above mentioned measurements to serve as valuable biomarkers for disease management in the future, following appropriate validation by clinical trials, as well as by large-scale real-world studies. Moreover, the panel agreed that given the variation in the time required for the full response and the various mechanisms of action that different DMTs exhibit, the evaluation of the therapeutic response towards a DMT requires the conduction of an MRI at least 3–6 months (re-baseline MRI) and at 1 year following DMT initiation. Topic II statements are presented in Table 2.

### 3.3. Topic III: Practice of Treatment Change (16 Statements)

The panel agreed that the distinction between 1st and 2nd line DMTs does not remain valid based on the recent literature. The panel agreed that the current concept in DMT classification based on efficacy is that DMTs in MS are categorized as moderate-efficacy (glatiramer acetate, interferon-β, teriflunomide and dimethyl fumarate) and high-efficacy DMTs (cladribine, natalizumab, fingolimod, ozanimod, ponesimod, siponimod, ocrelizumab, ublituximab, ofatumumab and alemtuzumab) [5,7,39]. However, in the question “is there convincing real-world evidence for such a concept, particularly on an individualized treatment approach?”, the panel did not reach consensus. Panel members argued that the current concept of different DMTs classification allows for earlier patient access to high-efficacy treatments, based on the treating physician’s suggestion and the appropriate indication based on the disease phenotype. Other scientific organizations have endorsed this [40] or a modified classification [9] for DMTs in MS based on their effect in reducing relapses, as evidenced by clinical trials. Panel members argued that more real-world evidence (RWE) studies are needed in order to support a broader consensus regarding DMTs classification. Clinicians who recommend a high-efficacy drug must be aware of the risks and the need of close monitoring of the patient. However, physicians should also take into account that the formulated question refers to an “individualized treatment approach”, thereby not referring to the comparison between different population groups. Panel members advocated that physicians should focus on a different direction in the MS field—i.e., for the management of the patient overall, taking into account the characteristics of the disease, patient’s preferences, comorbidities, safety, comparative studies’ results, etc.

The panel agreed that upon DMT switching due to lack of efficacy, one should prefer a DMT with a different mechanism of action. However, some considerations were formulated with respect to B-cell depleting agents, for which there are known differences in dose and mode of administration.

The panel did not reach a consensus regarding the statement “When issues related to safety, comorbidities and/or patient’s preferences are contemplated, a DMT switch within the therapeutic category of moderate-efficacy DMTs due to suboptimal response* (*defined as the presence of clinical and/or radiological activity) is reasonable” due to reasons of uncertainty in the formulation: as reasons for treatment switch include both comorbidities and the related safety issues, as well as clinical activity (relapses, etc.). As per core panel suggestion, the question was then divided into two parts. Following this, the panel agreed that in certain cases with suboptimal response to a moderate-efficacy DMT, a horizontal switch to another moderate-efficacy DMT may be a valid strategy. In order to facilitate the physicians’ decision, the panel notes on the Recommendations of the Hellenic Academy of Neuroimmunology [http://en.helani.gr/ (accessed on 19 May 2026)] endorsed by the Greek Ministry of Health, for the appropriate prescription of DMTs for MS. Available in: [https://www.moh.gov.gr/articles/health/domes-kai-draseis-gia-thn-ygeia/kwdikopoihseis/therapeytika-prwtokolla-syntagografhshs/diagnwstika-kai-therapeytika-prwtokolla-syntagografhshs/5414-diagnwstika-kai-therapeytika-prwtokolla-syntagografhshs-neyrologikw] (accessed on 19 May 2026) [41], according to which, in a patient with EDSS < 3, a lateral switch of DMTs may be favored in cases of no response. However, in cases with EDSS ≥ 3, therapy escalation should be preferred. Moreover, the panel agreed that, when issues related to safety, comorbidities and/or patient’s preferences are contemplated, a DMT switch within the therapeutic category of moderate-efficacy DMTs is reasonable.

The panel agreed that regardless of the previously observed clinical response, when issues related to adverse reactions, comorbid diseases/conditions and/or desire for pregnancy are contemplated, a switch from high- to moderate-efficacy DMTs should be considered (de-escalation).

The panel concluded that the close monitoring of PwMS under DMTs, presenting the progression of disability without disease activity, is needed. Therefore, according to data from the available studies up to now, the panel disagreed with the statement that “Treatment switch from moderate- to high-efficacy DMTs is a reasonable strategy upon the presence of confirmed progression of disability, also when evidence of disease activity is absent (escalation)”.

Cyclophosphamide and mitoxantrone have been previously used in refractory MS cases. Some people have benefited from these treatments, with regard to disability progression; however, in most studies, these agents were used in PwMS with high clinical disease and/or radiological activity [42,43,44]. Due to the associated risk of serious adverse events and due to the lack of robust data with regard to their impact on the progression of the disease, the panel concluded that this strategy should not be broadly applied. Therefore, all the members of the panel agreed that in most cases “upon the presence of confirmed progression of disability, also when evidence of disease activity is absent, off-label use of pharmaceutical agents in MS is a reasonable strategy. The decision should be made on the basis of clinical criteria, on a patient-specific basis and following the informed patient’s consent”.

The panel further discussed the long-term strategy for those PwMS who are initiated with a high-efficacy DMT. Members of the panel expressed their concerns about age-dependent safety issues, since according to the current literature, comorbidities [45], cancer [46] and serious infections [47] are more prevalent in aged PwMS. In addition, aging processes (immunosenescence and neurodegeneration) may further complicate disease management; disease activity declines while disease progression is often the main driver of disability, thus the effects of DMTs are minimized [47,48]. Members of the panel suggested that according to the recent evidence, patients over 55 years old, without activity in the past 5 years, could be discontinued from DMTs [8,49]. The concept of de-escalation was suggested, as an alternative strategy for cases with stable disease receiving a high-efficacy long-term DMT in the long term. Nevertheless, all panel members acknowledged that long-term real-world data for some DMTs demonstrate sufficient efficacy and safety; therefore, there is clearly a need for biomarkers and more data in order to identify those PwMS that would benefit from a long-term high-efficacy DMT. Therefore, the panel concluded that for PwMS under a high-efficacy DMT, maintenance of this a high-efficacy DMT strategy for the long-term is not always applicable. In addition, all members agreed that advancing age and absence of evidence of disease activity in the previous years may be factors to consider for the discontinuation of treatment with DMTs.

With regard to newly diagnosed PwMS with high disease activity, the panel agreed that in certain cases induction treatment with IRTs [50], followed by subsequent de-escalation with a moderate-efficacy DMT, may be considered as a valid option. In addition, the panel agreed that in certain cases, with clear signs of poor prognosis (such as male sex, cognitive impairment, multifocal signs at onset, short interval between first and second attack, incomplete remission after first relapses, early accumulation of disability), treatment initiation with high-efficacy DMTs is considered a valid strategy. Subsequent de-escalation could be considered as an option to those that present long-term remission of the disease under high-efficacy DMTs, especially when safety issues arise. Topic III statements are presented in Table 3. 

### 3.4. Topic IV: Reasons for Treatment Change

A need for treatment change may occur for various reasons. The panel reviewed statements about treatment change due to disease activity, cognitive deterioration, safety issues, pregnancy and adherence to treatment and/or monitoring schedule.

With regard to disease activity, the panel agreed that RAW should be prevented. All members agreed that a relapse that is associated with EDSS increase, irrespective of the degree of patient recovery, qualifies for a DMT switch. MRI activity, presented as new/enlarging T2 or T1 Gd+ lesions, also represents suboptimal response to treatment. Therefore, the panel concluded that MRI activity, in the absence of relapses and/or EDSS progression, can justify a DMT switch. Additionally, all members of the panel agreed that disease activity that is present for more than 1 year following DMT onset, even in the absence of EDSS increase, can qualify for a DMT switch. Overall, the panel concluded that any disease activity in PwMS treated with a DMT may justify a treatment change. However, such decisions should be performed taking into account that DMTs vary in the time needed to take full effect.

All members of the panel agreed that the existing evidence does not adequately support that cognitive impairment and the respective progression in cognitive impairment (quantified on the basis of a validated scale, as well as documenting alterations in at least two cognitive domains), in the absence of relapses or EDSS increase, can justify a treatment change. The panel concluded that, based on the current literature [51,52], cognitive rehabilitation may be proposed instead.

With regard to safety issues, the panel considers that all PwMS under DMTs should be periodically monitored for adverse events and laboratory abnormalities. All members of the panel agreed that a treatment switch should be considered in those cases where safety or tolerability issues arise from the use of a DMT. Therefore, the members of the panel stated that safety problems (adverse events, toxicity, laboratory abnormalities) can justify a DMT switch, even in patients without evidence of clinical and/or radiological activity. In addition, all members agreed that issues related to treatment tolerance can justify a DMT switch, even in patients without evidence of clinical and/or radiological activity. Members agreed that “when issues related to treatment tolerance are contemplated with a moderate-efficacy DMT, consider a DMT switch within the moderate-efficacy DMT group. In such cases, switch towards a high-efficacy DMT should be contemplated as a secondary option”.

The panel agreed that in most stable cases, irrespectively of previous levels of disease activity, where PwMS desire pregnancy, the switch to a DMT that is approved for pregnancy (interferon-β and glatiramer acetate) could be a valid option. More specifically, the members of the panel agreed that, when issues related to desire for pregnancy are contemplated, a modification of the therapeutic strategy in patients without current evidence of disease activity is reasonable even if disease control for this patient has previously been challenging. In addition, in a patient with previously very active disease, who achieved NEDA-3 and/or MEDA under a DMT that poses fetal risks and who desires to become pregnant, the panel proposes to consider a minor-risk DMT. In such cases the maintenance of the selected platform DMT, at least until pregnancy is confirmed, is a reasonable strategy. Finally, In a patient with previously mild/moderate disease activity, who achieved NEDA-3 and/or MEDA under a DMT that poses fetal risks and who desires to become pregnant, the switch to platform, low-risk DMT and maintenance of the selected platform DMT at least until pregnancy/lactation period is confirmed is a reasonable strategy. Topic IV statements are presented in Table 4.

**Figure 1:**
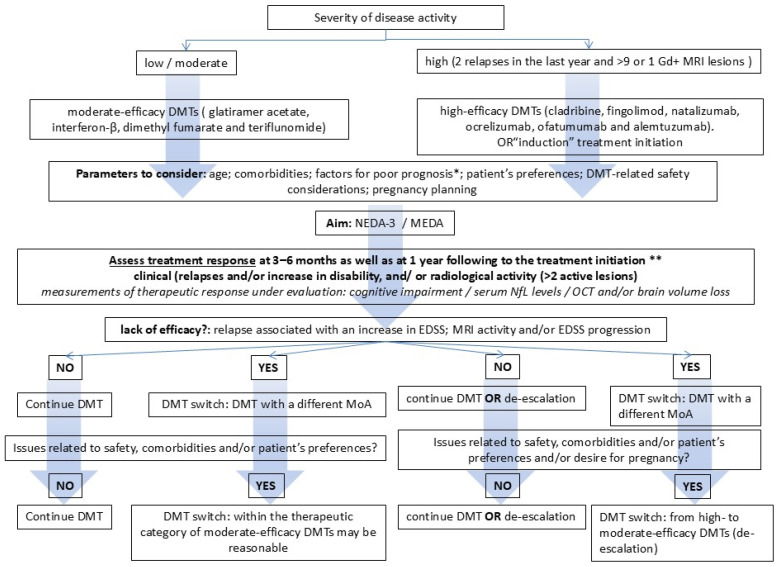
Proposed decision algorithm on DMT initial selection, treatment switch and de-escalation strategies. Gd, gadolinium; MRI, magnetic resonance imaging; DMT, disease-modifying treatment; NEDA, No Evidence of Disease Activity; MEDA, Minimal Evidence of Disease Activity; NfL, neurofilament light chain; OCT, Optical Coherence Tomography; EDSS, Expanded Disability Status Scale; MoA, mode of action. * Older age at disease onset, male, motor and/or cerebellar signs at onset, increased relapse rate in the years closely following disease onset, short interval between episodes, disability residual after the first few relapses, early accumulation of disability; ** a more extended period for cladribine (24 months).

Overall, the panel agreed that clinicians should discuss with PwMS about the potential dangers of non-adherence to medication and a monitoring plan. In the event of poor adherence to the treatment and/or monitoring plan, a treatment change to a DMT with less monitoring safety issues/monitoring burden could be an option, even in stable patients. This change may additionally be justified in the event of a lack of appropriate monitoring due to limited access to healthcare providers (MRI providers and laboratories for sera analyses). Therefore, the members of the panel concluded that a DMT switch in patients without evidence of disease activity due to lack of adherence is a reasonable strategy. Additionally, all members agreed that a DMT switch in patients without evidence of disease activity due to lack of appropriate monitoring is a reasonable strategy. A proposed decision algorithm based on different DMT initial selections and subsequent switch scenarios is summarized in Figure 1.

### 3.5. Topic V: Statements for Injectable Immunomodulatory Drugs and Bridging Therapy

The panel agreed that upon diagnosis, platform DMTs should be administered in patients with evidence of mild-to-moderate disease activity and in women with a desire for pregnancy in the short term [53]. A notification is being made for physicians to strictly agree with treatment recommendations for pregnancy in women with MS, with respect to the DMTs administered. The panel agreed that platform DMTs should be administered in patients with currently not-well-defined prognosis due to the evaluation of clinical or laboratory data being in progress shortly after disease onset. Moreover, there is strong real-world evidence regarding the safety of interferon-β and glatiramer acetate during pregnancy and breastfeeding [53,54]. Moreover, the panel agreed that physicians should prescribe an approved immunomodulatory therapy during pregnancy and the breastfeeding period when necessary. There is strong real-world evidence regarding the safety of injectable DMTs on cancer [55]. In PwMS that present with a history of previous cancer, an injectable DMT should be administered. The panel agreed that when issues related to acute/subacute cardiovascular and metabolic emergencies are contemplated in PwMS, DMT should be discontinued. The panel agreed that an extended assessment of possible infection risk at the time of diagnosis of MS and before treatment initiation, independent of treatment selection, should not be performed as a universal practice. However, the panel agreed that only when patients are under a certain DMT and only when a certain switch is considered, an extended assessment for possible infection risk should be performed [56]. As soon as the infection risk assessment is completed and positive for any latent or active infection, it is important to prescribe DMTs with no potential effects in either triggering or worsening the underlying infectious disease (i.e., immunomodulatory injectables). Regarding cases where high-efficacy DMTs have been selected in the first place, and while the immunization process is in progress, the panel agreed that bridging therapy with injectable DMTs may be administered, particularly in cases where treatment initiation should not be delayed.

The panel did not reach a consensus regarding whether physicians, when switching DMTs, should minimize the potential risks associated with a prolonged DMT washout period through the administration of injectable DMTs as bridging. In order to take into account cases of prolonged lymphopenia, hepatotoxicity or other adverse events due to treatments that may lead to prolonged treatment delay between treatment switches, this statement was rephrased by the core group. Following to this, the panel agreed that upon DMT switching, the risks associated with a prolonged treatment discontinuation/cessation should be minimized by the administration of bridging DMTs. Overall, most specialists agreed that the bridging therapy is a reasonable strategy in the clinical setting described above. However, the decision remains to be taken on a personalized basis, whereas, currently, evidence to support this approach based on large-scale real-world data is insufficient due to the lack of respective studies. In this respect, the panel agreed that currently there is not enough evidence that “bridging therapy” may be of any value. More clinical trials designed to address this question are needed. Topic V statements are presented in Table 5.

The panel agreed that the potential criteria for determining a bridging therapy cessation might be the following: recovery from side effects and pregnancy completion. The panel agreed that under certain circumstances (No Evidence of Disease Activity) the “bridging therapy” may be eventually retained for longer periods and thus considered as a maintenance therapy. The rationale is that this decision depicts a more medically oriented approach that aims to ameliorate as much as possible monitoring of the patients and their potential exposure to adverse events associated with treatments of higher efficacy. Concerns were expressed within the panel regarding the cessation of certain treatments known to be associated with rebound phenomena, such as natalizumab and/or fingolimod [57]. Of note, bridging therapy with platform treatments is not expected to ameliorate rebound phenomena. In this respect, the management of potential rebound is independent from—but does not exclude—bridging therapy, if bridging therapy is deemed necessary for reasons mentioned earlier in this chapter.

## 4. Discussion

The present study aimed to formulate a consensus, in order to provide expert recommendations on DMT switching and de-escalation strategies in Relapsing MS (RMS). Several DMTs are currently available for MS, aiming to prevent disease activity and disease-related disability [5,6,7,39]. Available MS treatments are substantially increased to include DMTs with different modes of action and to cover indications beyond RRMS. Based on the degree of their efficacy, DMTs for MS are commonly classified into DMTs of moderate-efficacy (including glatiramer acetate, interferon-β, teriflunomide and dimethyl fumarate) and DMTs of high-efficacy (cladribine, natalizumab, fingolimod, ozanimod, ponesimod, siponimod, ocrelizumab, ublituximab, ofatumumab and alemtuzumab). Treatment choice at any given time-point across the disease course should take into account several aspects, such as PwMS profile in terms of demographics, clinical characteristics of the disease, the possible presence of factors of poor or favorable prognosis, comorbidities, patients’ preference and lifestyle. With respect to the overall management of the disease in the era of several available DMTs, as well as treatment decision and the respective monitoring, several guidelines currently exist [5,7].

Due to the number of available treatments for MS, the complexity of decision-making has significantly increased over recent years. Currently, a large body of evidence supports two treatment approaches differentiated by the early use of DMTs of higher efficacy or not [58]. Early initiation of DMTs of higher efficacy was shown to exert a beneficial long-term effect on disease progression in MS, and the existing literature underlines the need for offering DMTs of higher efficacy early in the disease course [59,60,61,62,63,64,65].

Of note, disability worsening (EDSS change) in MS may come either as a result of RAW or as PIRA [35,66] and these two patterns of disability accumulation have been reported in all MS forms, including RRMS [34,67]. Moreover, NEDA-3 status is a goal of treatment in MS [68,69], as reaching and maintaining NEDA-3 status in the first 2 years following treatment initiation is reportedly linked with the absence of long-term disability in the following years [70]. The use of NEDA-3 has been widely applied in clinical trials that were designed to assess several of the more recently available DMTs, including the ones of higher efficacy, and has been considered as a useful method in order to target and monitor optimal treatment response in RRMS, especially in the absence of other available outcome measurements [71]. Importantly, whether NEDA-3 will be retained in the long term is also dependent on the disease activity prior to treatment initiation, as well as on the timing of said initiation, in addition to the degree of the DMT efficacy itself.

Although PIRA has been associated primarily with the underlying neurodegeneration in MS [33,34,35,72], it is not exclusively present in progressive MS [34,35]. PIRA has also been reported in patients at early stages of the disease, including patients with one demyelinating attack [73] and patients with early MS [33,35]. However, cohorts of patients with early MS exhibiting PIRA are largely understudied, thus posing challenges in defining their clinical and radiological characteristics. Moreover, the clinical implications of the early presence of PIRA for long-term disability are yet to be elucidated. Taking into account that PIRA depicts a neurodegenerative disease component in clinical terms, for patients with a first PIRA event early in the disease course it is reasonable to consider whether this event is associated with a particular outcome in terms of long-term prognosis. Additionally, one should also consider the fact that little is known about the association of PIRA with neuroinflammation, whether acute or chronic [33,35]. Furthermore, the definition and confirmation of PIRA in a clinical setting is still under investigation [74,75]. Disability accumulation in the absence of relapses may be reversible or not confirmed in a follow-up visit [75]. EDSS may be unable to detect subtle changes; additional modalities such as advanced neuroimaging, OCT and serum biomarkers may be needed in order to identify people at risk for developing PIRA [74,76]. In this context, machine learning approaches trained in large clinical datasets may soon aid clinicians in the prediction of future disability progression [77].

With respect to clinical trials, PIRA is a newly introduced term in the field. For some of the drugs known to target disease activity, clinical trials indicate that they may reduce PIRA as well. PIRA may be evident in a patient with remitting disease. Pivotal clinical trials have been designed on the basis of the distinction of relapsing–remitting and/or progressive disease, as formulated in [78]; however, recent reports challenge this distinction [34,67]. In this respect, to date, a beneficial effect on PIRA was shown in several DMTs, though to a various extent depending on study, ocrelizumab and ofatumumab, as well as natalizumab [35,79,80,81,82,83,84]. In most comparative studies, PIRA incidence was found to be lower in people treated with high-efficacy DMTs, as compared with people treated with moderate-efficacy DMTs [35,77,85,86]. Nevertheless, some studies failed to demonstrate this superiority of high-efficacy DMTs [83,87,88]. To date, there are no data that support the superiority of one high-efficacy DMT vs. another high-efficacy DMT with respect to PIRA. Therefore, more studies using standardized methodologies for PIRA detection and quantification are needed in order to determine the actual impact of various DMTs in disability progression.

Regarding Topic II (suboptimal response criteria—definitions), a universally accepted definition of suboptimal response to DMT in PwMS is currently not available. Thus, the decision on the timing and the exact practice of DMT switching poses special challenges. On the other hand, a timely treatment switch for patients who do not exhibit optimal response to a DMT is crucial, as it poses implications for long-term clinical outcomes. Upon suboptimal response, physicians may suggest switching to another DMT of similar efficacy or to another DMT of higher efficacy. According to the regulating authorities in Europe, for treatments of higher efficacy, such as fingolimod and natalizumab, administration should follow previous treatment failure. In this respect, eligible patients should have exhibited ≥1 relapse in the previous year while receiving the previous therapy and/or have ≥9 T2 lesions or ≥1 Gd+ lesion on brain MRI. Other definitions for suboptimal response to a DMT include stable or increased relapse rate and/or ongoing severe relapses in the year prior to the DMT switch [89,90]. Other definitions of suboptimal response include relapse frequency, disease progression (measured by the EDSS) and MRI activity characteristics [91,92]. Early studies on assessing the suboptimal response to DMTs underline the necessity to assess MRI activity regularly during DMT administration and to timely consider treatment modification, at the event of clinical and/or MRI activity (the later, in some cases even in the absence of relapses, depending on the relative burden of the MRI activity) [93,94]. The need for regular clinical and MRI monitoring has also been underlined in recent guidelines and remains, even in the recently evolved landscape of DMTs in MS [5,95]. Additional definitions of radiological evidence of suboptimal response to DMTs include the presence of ≥3 new/enlarged T2 lesions and/or ≥2 Gd+ T1 lesions, although a universally accepted number of new T2 lesions that qualify for breakthrough disease activity on MRI is lacking [28,96,97,98].

Although the panel agreed that at present there is not enough evidence for cognitive impairment/serum NfL levels/OCT and/or brain volume loss to be regarded as validated measurements of therapeutic response, upon open discussion, the potential of these measurements to guide clinical decision-making in the future, following appropriate validation, has been acknowledged. Recent evidence highlights the association between increased serum GFAP and the presence of PIRA, as well as between serum NfL and inflammatory disease activity, thus posing these biomarkers as significant candidates for clinical decision-making in the future, following large-scale validation [99]. Importantly, RNFL thickness evaluation via OCT is a biomarker that has recently been incorporated into the clinical care for MS [20,100]. Further evidence links RNFL thickness with the risk of disease progression and sustained disability accumulation [101], as well as with brain and spinal cord atrophy [102]. In this respect, OCT is a tool that poses potential implication of prognostic value for MS, again following extensive validation. Of note, brain atrophy associates with cognitive decline in the long term, and measurements of brain volume and cognition have been the subject of extensive research in order to better characterize their dynamic association and their potential to guide as biomarkers in clinical decision-making [103].

Regarding Topic III (strategies for treatment change), physicians should take into account that moderate-efficacy DMTs may achieve long-term disease remission in certain patients. Clinical trials with a direct comparison among these DMTs are scarce, with the vast majority of clinical trials for a new DMT under evaluation using IFN-b as a comparator drug with well-described efficacy and well-known safety profile [104,105,106]. Regarding treatment switch, several registry-based studies provide evidence that may guide clinical decisions, especially when taking into account the broad spectrum of treatment choices available over the last decade [107]. One aspect that warrants a clinical decision is the choice between a treatment switch within treatments of moderate efficacy (horizontal switching) or from a treatment of moderate efficacy to a treatment of higher efficacy (vertical switching/treatment escalation). Existing evidence indicates that the latter should be preferred for patients with active/breakthrough disease and/or with evident accumulation of disability, whereas a subset of patients with lower disability may be eligible for horizontal switching, especially when the treatment change is contemplated in the setting of adverse events and/or patients’ preferences [108,109,110]. However, with respect to horizontal switching, a strong recommendation is currently lacking. Regarding the patterns of treatment sequential choices, real-world data are favoring switching directly to a high-efficacy treatment, compared to a more step-wise approach [63,111]. Moreover, registry-based data on the patient- and disease-related factors that predict treatment switches indicate that the early initiation of highly active treatment may be beneficial compared to the more widely used escalation strategy; however, currently, further studies that will evaluate this assumption are needed [112,113].

Regarding the selection of a DMT with a different mechanism of action, special reference is made on B-cell targeting treatments: ofatumumab is a fully human antibody that induces a ~50% reduction in B-cells, whereas ocrelizumab is a humanized antibody that eliminates B-cells. Ofatumumab is expected to induce fewer neutralizing antibodies compared to ocrelizumab and rituximab. Moreover, for a patient under ofatumumab who transitions to progressive disease, a switch to ocrelizumab may be justifiable, as there is clinical evidence available that ocrelizumab is efficient in progressive active forms of the disease. The panel agreed that there is no controversy between this and the moderate/high-efficacy treatment concept.

Recently, de-escalation strategies are the focus of extensive research. Strategies advocated for this purpose include extended interval dosing, switching from high-efficacy DMTs to DMTs of moderate efficacy, and IRTs [114]. For IRTs, their mechanism of action has been proposed as an inherent de-escalation strategy, as these DMTs have been shown to induce prolonged disease remission without the need for additional maintenance treatment [115]. Another approach is treatment discontinuation, although not a de-escalation strategy per se, it has also been addressed in the context of de-escalation. More specifically, treatment discontinuation in older PwMS and in patients aged over 18 has been studied in the frame of DISCOMS and DOT-MS studies, respectively, showing lower incidence of recurrent inflammatory disease activity following treatment discontinuation in older patients who have been stable for over 5 years [49,116]. Similar findings were provided by a recent meta-analysis, where stable patients over 50 years old did not have any significant increase in relapses after discontinuing treatment [117] (for a list of recent studies which examined the effects of DMT discontinuation on disability please see [115]). Discontinuation of DMTs in PwMS with documented progression without signs of inflammatory activity, especially in those who are non-ambulatory and/or over 60 years old may be a reasonable scenario [115].

De-escalation strategies have also been sought following the discontinuation of high-efficacy DMTs. Several previous studies have addressed the strategy of switching from natalizumab, upon high JC virus (JCV) index, towards fingolimod, as this approach prevented post-natalizumab relapse risk, especially when the switch was concluded within 8 weeks of the natalizumab washout period [118,119]. A short washout period of one month when switching from natalizumab has also been advocated [120]. Of note, anti-CD20 DMTs have shown a favorable profile compared to fingolimod and dimethyl fumarate, in terms of safety and of clinical outcomes of recurrent disease activity upon switching from natalizumab, thus posing anti-CD-20 DMTs as a reasonable treatment approach following natalizumab cessation [121,122].

Overall, treatment de-escalation is not a unified approach and precise evidence to guide clinical decisions is currently lacking. Large real-world observational studies have been implemented in order to provide further insight. A large multi-center observational study examining adult patients with RRMS who underwent treatment de-escalation stratified as switching from high- to medium-, high- to low-, or medium- to low-efficacy treatment and comparing outcomes with patients that continued the initial DMT highlighted the need for individualized treatment decisions [123]. Main factors to consider are the age of treatment de-escalation, as well as the presence of disease activity in the previous years, factors that pose a significant impact on the risk for disease activity following treatment de-escalation [124,125]. It should be noted that although inflammatory activity is expected to decrease with age, older adults are more likely to present PIRA [88]. However, evidence regarding the ability of DMTs to prevent PIRA in the aging MS population remains insufficient. A group of experts, organized by ECTRIMS, has recently proposed algorithms and scenarios for the use of a de-escalating strategy [115]. The experts suggest that in cases of aged PwMS with at least 5 years of stable disease, a de-escalation strategy could be considered. Additionally, the risk of cancer, prolonged low IgG levels under anti-CD20 treatment, pregnancy planning, lymphopenia, hepatotoxicity and comorbid conditions may constitute reasons for a DMT de-escalation [115,126].

Regarding Topic IV (reasons for treatment change), special reference is being made to cognitive deterioration. Current studies suggest that cognitive performance may also worsen during disease activity; in this case a partial recovery is expected during the next months [127], and, therefore, a re-baseline of cognitive performance 3 to 6 months following clinical activity may be needed [128]. Pregnancy sets additional challenges in the management of MS, since many DMTs may pose serious fetal risks. A desire for pregnancy should be followed by a specific treatment plan [129,130]. Poor adherence to treatment may negatively affect the disease course while non-adherence to a monitoring schedule poses serious risks for adverse events [131].

Regarding Topic V (statements for injectable immunomodulatory drugs and bridging therapy), the panel accepted the term “bridging therapy” as a therapy used during the transitional period between different treatments or when a primary treatment temporarily is not prescribed due to other, usually health-related, reasons. Examples of this concept include transplantation procedures, as well as anticoagulant treatment (e.g., bridging with heparin) [132,133]. Immune therapies may also be used in a similar context, for instance, therapeutic plasma exchange and intravenous immunoglobulins are frequently used upon slower-acting, immunosuppressive treatment initiation for myasthenia gravis.

In PwMS, bridging therapies have been advocated for use upon switching between treatments. Usually, injectable immunomodulatory drugs (namely, interferon or glatiramer acetate) are administered as bridging treatments with a well-described efficacy, safety and tolerability profile, and they are also well-suited for fragile patients. Of note, past and ongoing clinical trials have investigated treatment switches [134,135]; however, the bridging approach in MS has not been adequately addressed [136]. Due to the possibility of rebound phenomena with certain medications (such as fingolimod and natalizumab), a bridging strategy could be applied in cases of family planning and drug-related adverse events (such as lymphopenia and hepatotoxicity), especially in PwMS with recent inflammatory activity [115]. In certain patients, after providing a bridging therapy [126], the possibility exists that the patient does not exhibit adverse events and/or pregnancy does not occur. At the event that a patient also does not exhibit disease activity, then the bridging therapy may be continued further and thus considered as maintenance therapy. An additional clarification is hereby being made regarding the need for bridging upon lymphopenia. Physicians should be aware of the fact that only treatment cessation due to lymphopenia as an adverse event and prolonged subsequent lymphopenia would warrant the need for a bridging treatment, whereas lymphopenia associated with a known drug’s mode of action would not qualify for treatment bridging. Further, physicians should consider the fact that disease activity may not constantly be high across the disease’s overall duration; rather, a patient once evaluated as exhibiting high disease activity may subsequently be re-evaluated as exhibiting mild to moderate disease activity. Factors such as aging/immune senescence or epitope spreading may modify the disease underlying pathology and thus have an effect on the disease phenotype. In this respect, the continuation of a platform treatment, initially administered as a bridging therapy, also in a maintenance setting in carefully selected individuals is a reasonable approach.

## 5. Conclusions

Overall, current existing clinical tools do not allow for a concrete definition/clinical setting of bridging treatment in MS. However, close monitoring for re-emerging disease activity in a patient who receives platform treatment for a prolonged time period (originally initiated as “bridging”), provides a clinical setting that a) prevents patients’ exposure to potential adverse events associated with treatments for highly active disease and b) allows for “active surveillance” of the disease activity and increased alertness from the physician to re-evaluate and timely adjust treatment decisions. In the absence of adequate evidence to guide treatment decisions on a personalized basis, further large-scale studies are needed.

The therapeutic landscape of MS is still expanding. Numerous DMTs with different mechanisms of action exist while others are currently under development. Current research about DMT choice, switch or de-escalation remains limited. Therefore, the statements produced by this panel should not be considered as guidelines that can be uniformly adapted to all PwMS, but rather an expert opinion that may help clinicians to form their clinical decision. In addition, due to the fact that the present study depicts a consensus of a relatively small number of experts from a single country, thus introducing potential selection bias, caution is warranted regarding the generalizability of the findings. The present consensus may be subjected in limitations owing to local prescribing practices, healthcare system factors and national guidelines. In accordance with the widely accepted consensus, however, the choice of treatment should still be based on an individualized approach, taking into account inflammatory activity and factors such as patient preferences, comorbid conditions and age. Novel biomarkers may soon help clinicians predict future disability accumulation, but, for the meantime, regular and detailed patient monitoring seems to be the optimal way to guide clinicians’ decisions on treatment changes.

## Figures and Tables

**Table 1:** Statements on Chapter I (treatment aims) and respective consensus status.

Evidence/Opinion Based	Statement	Consensus 1st Round	Consensus 2nd Round
evidence based	**The selection of first-choice treatment, as well as the following ones, is largely based on the clinical and radiological characteristics of the disease (disease activity: relapses and/or presence of MRI lesions).**	**100% YES**	**100% YES**
N/A	**Definition of relapse/pseudo-relapse (*refer to text*).**	**100% YES**	**100% YES**
evidence based	**The selection of treatment** largely takes into account the severity of disease activity (low/moderate/high).	YES	YES
evidence based	‘High activity’, defined in several SPCs of high-efficacy DMTs (≥2 relapses in the last year, ≥1 Gd+ brain MRI lesions or new/enlarged T2 lesions), does not accurately depict the current clinical practice and must be revised.	YES	YES
evidence based	**The treatment aim, defined as control of disease activity as means to prevent relapses, disability worsening and/or disability progression, must be universally applied for all DMTs (first-/second-choice treatment, etc.).**	**100% YES**	**100% YES**
evidence based	**NEDA-3 is highly desirable; however, it is frequently difficult to reach and to maintain over the disease course.**	**100% YES**	**100% YES**
opinion based	MEDA is more frequently reached and, therefore, it constituted a more attainable aim than NEDA in the assessment of optimal treatment response.	YES	YES
opinion based	**A possible universally applied definition for MEDA may be the absence of relapses and/or disability progression; however, ≤two MRI lesions (Rio score 0)] are typically permitted.**	**100% YES**	**100% YES**
evidence based	**Early initiation of treatment definition/necessity.**	**100% YES**	**100% YES**
evidence based	**DMT initiation in a treatment-naïve elderly patient free of clinical and/or MRI disease activity is not recommended.**	**100% YES**	**100% YES**
evidence based	**A DMT therapeutic window is a reasonable time period that a patient should undergo under a specific DMT prior to the assessment of DMT efficacy and the potential consideration of treatment switch).**	**100% YES**	**100% YES**
evidence based	Parameters to consider the “therapeutic window” for each DMT necessary to provide individualized decision-making and also to include other measures such as the number and location of MRI active lesions and/or relapses during the “therapeutic window period”.	No consensus—rephrased	YES

MRI, magnetic resonance imaging; SPC, summary of product characteristics; DMTs, disease-modifying therapies; NEDA, No Evidence of Disease Activity; MEDA, Minimal Evidence of Disease Activity; Gd+, gadolinium enhancing; bold indicates statements with 100% consensus.

**Table 2:** Statements on Chapter II (suboptimal treatment response) and respective consensus status.

Evidence/Opinion Based	Statement	Consensus 1st Round	Consensus 2nd Round
evidence based	**There is not a set definition for DMT suboptimal response; however, consideration should be given to the clinical (relapses and/or increase in disability), relapse-depended or independent, and/or radiological activity (>2 active lesions) characteristics.**	**100% YES**	**100% YES**
evidence based	**DMT suboptimal response is a term applicable for all DMTs over the course of the disease; however, initial evaluation should be conducted 6–12 months following to the treatment onset *(a special reference on cladribine was added in the second round).***	**100% YES**	**100% YES**
evidence based	A more extended period for cladribine (24 months). During the first year with cladribine treatment a patient may have disease activity; however, the treatment is regarded as completed after the second course (2nd year).	N/A	**100% YES**
evidence based	**At present there is not enough evidence for cognitive impairment/serum NfL levels/OCT and/or brain volume loss to be regarded as validated measurements of therapeutic response.**	**100% YES**	**100% YES**
evidence based	**By taking into account the various minimal time for response and the mode of action for different DMTs, an MRI assessment should be conducted for the treatment response evaluation at 3–6 months, as well as at 1 year following to the treatment initiation.**	**100% YES**	**100% YES**

MRI, magnetic resonance imaging; DMTs, disease-modifying therapies; OCT, Optical Coherence Tomography; NfL, neurofilament light chain; bold indicates statements with 100% consensus.

**Table 3:** Statements on Chapter III (practice of treatment change) and respective consensus status.

Evidence/Opinion Based	Statement	Consensus 1st Round	Consensus 2nd Round
evidence based	The current concept of different DMTs classification based on their efficacy identifies: (1) moderate-efficacy DMTs (namely, glatiramer acetate, interferon-β, dimethyl fumarate and teriflunomide) and (2) high-efficacy DMTs (namely, cladribine, natalizumab, fingolimod, ozanimod, ponesimod, siponimod, ocrelizumab, ublituximab, ofatumumab and alemtuzumab). Is there convincing real-world evidence for such a concept, particularly on an individualized treatment approach?	YES	YES
evidence	Although the distinction between moderate- and high-efficacy DMTs is universally accepted, the choice of DMT depends on the neurologists’ opinion, after taking into account the characteristics of the disease, patient’s preferences, as well as DMT-related safety considerations.	YES	YES
evidence based	Upon DMT switching due to lack of efficacy, one should prefer a DMT with a different mechanism of action. Is this concept still valid?	N/A	YES
evidence based	Upon DMT switching due to lack of efficacy, one should prefer a DMT with a different mechanism of action. Is there any controversy between this and the moderate/high-efficacy treatment concept?	N/A	YES
evidence based	Upon DMT switching due to safety reasons, one should prefer a DMT with a different mechanism of action.	YES	YES
evidence based	In certain cases, a DMT switch within the therapeutic category of moderate-efficacy DMTs due to suboptimal response (defined as the presence of clinical and/or radiological activity) is reasonable.	N/A	YES
evidence based	When issues related to safety, comorbidities and/or patient’s preferences are contemplated, a DMT switch within the therapeutic category of moderate-efficacy DMTs is reasonable.	N/A	YES
**evidence based**	**Regardless of the previously observed clinical response, when issues related to adverse reactions, comorbid diseases/conditions and/or desire for pregnancy are contemplated, a switch from high- to moderate-efficacy DMTs should be considered (de-escalation).**	**100% YES**	**100% YES**
evidence based	Discontinuation of DMTs is reasonable to consider after taking into account advancing age and absence of evidence of disease activity in the previous years.	YES	YES
evidence based	In certain cases, with “induction” treatment initiation (with alemtuzumab or cladribine) (upon high activity: two relapses in the last year and >9 or 1 Gd+ MRI lesions), de-escalation is a reasonable strategy, regardless of current activity level.	100% YES	100% YES
**evidence based**	**In certain cases, when factors of poor prognosis are present, treatment initiation with high-efficacy DMTs followed by de-escalation is a reasonable strategy (factors for poor prognosis in MS: older age at disease onset, male, motor and/or cerebellar signs at onset, increased relapse rate in the years closely following disease onset, short interval between episodes, disability residual after the first few relapses, early accumulation of disability).**	**100% YES**	**100% YES**

MRI, magnetic resonance imaging; DMTs, disease-modifying therapies; Gd+, gadolinium enhancing; bold indicates statements with 100% consensus.

**Table 4:** Statements on Chapter IV (reasons for treatment change) and respective consensus status.

Evidence/Opinion Based	Statement	Consensus 1st Round	Consensus 2nd Round
evidence based	Regarding the presence of one relapse as justification for treatment switch: time of occurrence in the frame of treatment initiation, severity, functional changes.	100% YES	100% YES
evidence based	A relapse associated with an increase in EDSS, irrespective of the degree of residual and/or patient recovery, can justify a DMT change switch.	100% YES	100% YES
evidence based	MRI activity (new/enlarging T2/Gd+ enhancing lesions) in the absence of relapses and/or EDSS progression can justify a DMT change switch.	100% YES	100% YES
evidence based	Clinical and/or MRI activity that is present for more than 1 year following to DMT initiation, even in the absence of EDSS increase, can justify a DMT switch (also when taking into account the MRI activity and/or the severity of relapses, irrespective of the degree of patient recovery).	100% YES	100% YES
evidence based	Safety issues, such as adverse events and/or laboratory abnormalities) can justify DMT switch, also in patients free of clinical and/or radiological activity.	100% YES	100% YES
evidence based	Issues related to treatment tolerance can justify DMT switch, also in patients free of clinical and/or radiological activity.	100% YES	100% YES
evidence based	When issues related to treatment tolerance are contemplated with a moderate-efficacy DMT, consider a DMT switch within the moderate-efficacy DMT group. In such cases, a switch towards a high-efficacy DMT should be contemplated as a secondary option.	YES	YES
evidence based	When issues related to desire for pregnancy are contemplated, a modification of the therapeutic strategy in patients without current evidence of disease activity is reasonable even if disease control for this patient has previously been challenging.	100% YES	100% YES
evidence based	When issues related to desire for pregnancy are contemplated in a patient with previously very active disease, who achieved NEDA-3 and/or MEDA under a DMT that poses fetal risks, such as cladribine, natalizumab, fingolimod, alemtuzumab, ocrelizumab or ofatumumab, a DMT switch towards a high-efficacy DMT administered in the short term is reasonable.	YES	YES
evidence based	In a patient with previously very active disease, who achieved NEDA-3 and/or MEDA under a DMT that poses fetal risks and who desires to become pregnant, a switch to platform, low-risk DMTs and maintenance of the selected platform DMT at least until pregnancy is confirmed is a reasonable strategy.	100% YES	100% YES
evidence based	In a patient with previously mild/moderate disease activity, who achieved NEDA-3 and/or MEDA under a DMT that poses fetal risks and who desires to become pregnant, a switch to platform, low-risk DMT and maintenance of the selected platform DMT at least until pregnancy/lactation period is confirmed is a reasonable strategy.	100% YES	100% YES
evidence based	DMT switch in patients free of clinical and/or radiological activity due to lack of adherence is a reasonable strategy (PSPs could benefit poor adherence).	100% YES	100% YES
evidence based	DMT switch in patients free of clinical and/or radiological activity due to lack of appropriate monitoring is a reasonable strategy.	100% YES	100% YES

MRI, magnetic resonance imaging; DMTs, disease-modifying therapies; NEDA, No Evidence of Disease Activity; MEDA, Minimal Evidence of Disease Activity; EDSS, Expanded Disability Status Scale; Gd+, gadolinium enhancing.

**Table 5:** Statements on Chapter V (injectable immunomodulatory drugs and bridging therapy) and respective consensus status.

Evidence/Opinion Based	Statement	Consensus 1st Round	Consensus 2nd Round
evidence based	Upon diagnosis, platform DMTs should be administered in patients with evidence of mild-to-moderate disease activity and in women with a desire for pregnancy in the short term.	YES	YES
evidence based	Platform DMTs should be administered in patients with currently not-well-defined prognosis due to the evaluation of clinical or laboratory data being in progress, shortly after disease onset.	YES	YES
evidence based	There is strong real-world evidence regarding the safety of interferon-β and glatiramer acetate during pregnancy and breastfeeding.	YES	YES
evidence based	Prescribe an approved immunomodulatory therapy during pregnancy and breastfeeding period when necessary.	YES	YES
evidence based	There is strong real-world evidence regarding the safety of injectable DMTs on cancer.	YES	YES
evidence based	In PwMS that present with a history of previous cancer, an injectable DMT should be administered.	YES	YES
evidence based	When issues related to acute/subacute cardiovascular and metabolic emergencies are contemplated in PwMS, DMT should be discontinued.	YES	YES
evidence based	In patients under certain DMTs and when a certain DMT switch is considered, an extended assessment for possible infection risk should be conducted.	YES	YES
evidence based	As soon as the infection risk assessment is completed and positive for any latent or active infection, it is important to prescribe DMTs with no potential effects in either triggering or worsening the underlying infectious disease (i.e., immunomodulatory injectables).	YES	YES
evidence based	In cases where high-efficacy DMTs have been selected in the first place and while the immunization process is in progress, bridging therapy with injectable DMTs is appropriate, particularly in cases where treatment initiation should not be delayed	YES	YES
opinion based	Upon DMT switching, the risks associated with a prolonged treatment discontinuation/cessation should be minimized by the administration of bridging DMTs.	YES	YES
opinion based	The potential criteria for determining bridging therapy cessation might be the following: recovery from side effects and pregnancy completion. Do you agree?	YES	YES
opinion based	May under certain circumstances (No Evidence of Disease Activity) the “bridging therapy” be considered as a maintenance therapy at the end?	YES	YES

DMTs, disease-modifying therapies.

## Data Availability

No new data were created.

## Supplementary Materials

The following supporting information can be downloaded at: https://www.mdpi.com/article/10.3390/brainsci16060580/s1, Table S1: Rejected or negatively agreed statements.

## Abbreviations

The following abbreviations are used in this manuscript:

DMT	Disease-modifying treatment
EDSS	Expanded Disability Status Scale
Gd+	Gadolinium enhancing
IRT	Immune reconstitution treatment
MRI	Magnetic resonance imaging
MEDA	Minimal Evidence of Disease Activity
MS	Multiple Sclerosis
NfL	Neurofilament light chain
NEDA	No Evidence of Disease Activity
OCT	Optical Coherence Tomography
PwMS	People with Multiple Sclerosis
PIRA	Progression Independent of Relapse Activity
RAW	RelapseAssociated Worsening
RMS	Relapsing Multiple Sclerosis
RNFL	Retinal nerve fiber layer
RRMS	Relapsing–Remitting Multiple Sclerosis
RWE	Real-world evidence
SPC	Summary of product characteristics
