# Supplementary material for: Building Towards Initiation, Moderation, De-Escalation and Cessation of Disease-Modifying Treatments for Multiple Sclerosis in Greece: An Expert Panel Consensus Meeting
Source: Brain Sci. 2026 May 29;16(6):580. doi: 10.3390/brainsci16060580 (PMC13297107; doi:10.3390/brainsci16060580)
Supplement: Supplementary file 1 [file brainsci-16-00580-s001.zip › brainsci-4269732-supplementary.pdf]

Supplementary Table S1. Rejected or negatively agreed statements

Evidence / Opinion – based	Statement	Consensus first round	Consensus second round
**Chapter I: Treatment aims**			
Opinion – based	PIRA is a reasonable and important clinical outcome to consider for treatment choice	NO (negative consensus)	NO (negative consensus)
**Chapter III: Practice of treatment change**			
Evidence – based	Does the distinction between 1st and 2nd line DMTs remain valid?	NO (negative consensus)	NO (negative consensus)
N/A	Upon DMT switching due to lack of efficacy, one should prefer a DMT with a different mechanism of action. Is this concept still valid? Is there any controversy between this and the moderate/high-efficacy treatment concept?	No consensus - separated into 2 parts	N/A
N/A	When issues related to safety, comorbidities and/or patient's preferences are contemplated, a DMT switch within the therapeutic category of moderate-efficacy DMTs due to suboptimal response* (* defined as the presence of clinical and/or radiological activity) is reasonable	No consensus - separated into 2 parts	N/A
N/A	Treatment switch from moderate- to high-efficacy DMTs is a reasonable strategy upon the presence of confirmed progression of disability, also when evidence of disease activity is absent (escalation).	No (did not reach consensus)	N/A
N/A	Upon the presence of confirmed progression of disability, also when evidence of disease activity is absent, off-label use of pharmaceutical agents in MS is a reasonable strategy. The decision should be made on the basis of clinical criteria, on a patient-specific basis and following to the informed patient's consent.	No (did not reach consensus)	N/A
N/A	Overall, is there enough evidence to support the strategy according to witch, high-efficacy DMT once initiated, should be maintained in the long-term? (For how long / the de-escalation concept / the need of biomarkers)	No (did not reach consensus)	N/A
**Chapter IV: Reasons for treatment change**			
Evidence – based	Existing evidence does not adequately support the the presence of cognitive impairment and the respective progression in cognitive impairment (quantified on the basis of a	100% NO (negative	100% NO (negative

	validated scale, as well as documenting alterations in at least two cognitive domains), in the absence of relapses or EDSS increase can justify a DMT switch (cognitive rehabilitation is suggested).	consensus)	consensus)
**Chapter V: Statements for injectable immunomodulatory drugs and bridging therapy**			
Evidence – based	Upon MS diagnosis and prior to treatment initiation, independent of DMT, an extended assessment for infection risk should be conducted.	NO (negative consensus)	NO (negative consensus)
Opinion – based	Is there enough evidence that "bridging therapy" may be of any value? - no	NO (negative consensus)	NO (negative consensus)

DMTs Disease modifying therapies; MS Multiple Sclerosis; EDSS Expanded Disability Status Scale
